# Modeling of Root Nitrate Responses Suggests Preferential Foraging Arises From the Integration of Demand, Supply and Local Presence Signals

**DOI:** 10.3389/fpls.2020.00708

**Published:** 2020-05-27

**Authors:** Meine D. Boer, Joana Santos Teixeira, Kirsten H. Ten Tusscher

**Affiliations:** Computational Developmental Biology Group, Utrecht University, Utrecht, Netherlands

**Keywords:** phenotypic plasticity, preferential root nitrate foraging, modeling, demand and supply signals

## Abstract

A plants’ fitness to a large extent depends on its capacity to adapt to spatio-temporally varying environmental conditions. One such environmental condition to which plants display extensive phenotypic plasticity is soil nitrate levels and patterns. In response to heterogeneous nitrate distribution, plants show a so-called preferential foraging response. Herein root growth is enhanced in high nitrate patches and repressed in low nitrate locations beyond a level that can be explained from local nitrate sensing. Although various molecular players involved in this preferential foraging behavior have been identified, how these together shape root system adaptation has remained unresolved. Here we use a simple modeling approach in which we incrementally incorporate the known molecular pathways to investigate the combination of regulatory mechanisms that underly preferential root nitrate foraging. Our model suggests that instead of involving a growth suppressing supply signal, growth reduction on the low nitrate side may arise from reduced root foraging and increased competition for carbon. Additionally, our work suggests that the long distance CK signaling involved in preferential root foraging may function as a supply signal modulating demand signaling strength. We illustrate how this integration of demand and supply signals prevents excessive preferential foraging under conditions in which demand is not met by sufficient supply and a more generic foraging in search of nitrate should be maintained.

## Introduction

Phenotypic plasticity is of critical importance for sessile plants to adapt to and survive in a variable, heterogeneous environment. One of the environmental factors to which the root system of plants display extensive phenotypic variation is soil nitrate availability. Adaptation to spatio-temporally variable nitrate availability entails changes in nitrate storage and assimilation, adjustment in the spatial patterns, types, numbers, and affinity of expressed nitrate transporters as well as extensive adjustment of overall root system architecture (RSA) (Aibara and Miwa, 2014). Harnessing the full range of this plasticity may reduce the demand for artificial fertilizers and improve agriculture on poor soils, yet requires an improved understanding of the processes underlying this plasticity. This improved understanding is also needed to help combat the deleterious effects of excess nitrate deposition on natural ecosystem diversity.

**FIGURE 1:**
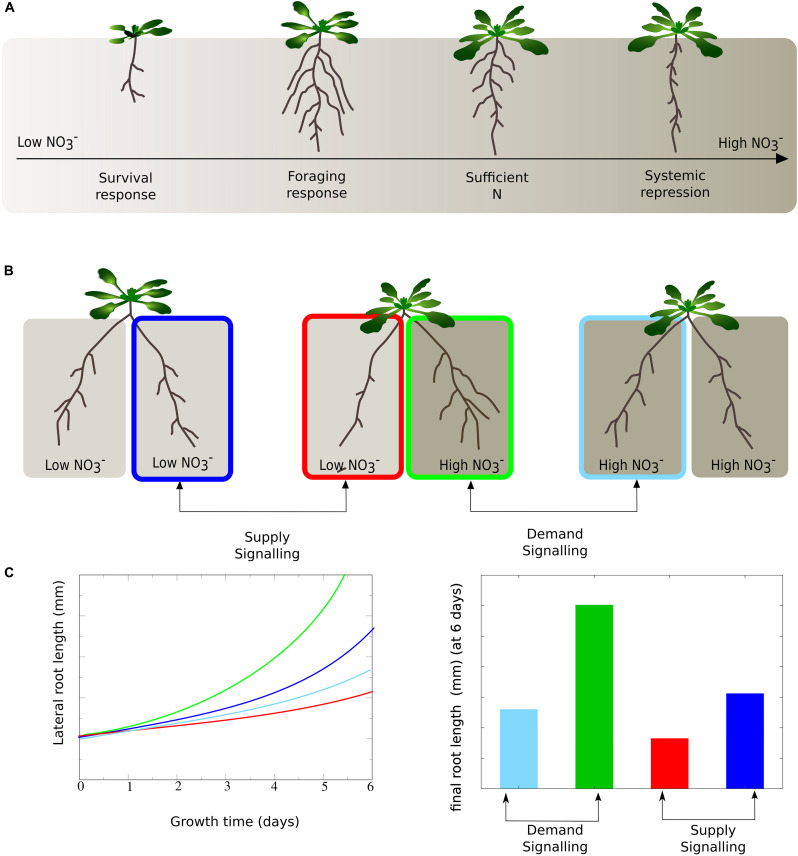
Response of root system architecture to environmental and internal nitrate status. **(A)** Range of growth responses occurring for increasing homogeneous external nitrate levels. For the lowest nitrate levels a growth repressing survival response occurs, somewhat less low nitrate levels induce a growth promoting foraging response, whereas very high nitrate levels induce a growth reducing systemic repression response. **(B)** Response of root growth in a split root architecture to homogeneous or heterogeneous nitrate conditions. Enhanced root growth at the high nitrate side under heterogeneous as compared to homogeneous conditions is indicative of the presence of a growth promoting demand signal. Similarly, reduced growth at the low nitrate side under heterogeneous as compared to homogeneous conditions indicates a growth repressing supply signal. **(C)** Schematized depiction of typical cumulative lateral root length growth dynamics (left) and final size after 6 days (right) at one side of the root system for indicated experimental conditions; curves and bar plots based on observations reported by Ruffel et al. (2011); Guan et al. (2014); Mounier et al. (2014).

Because of the extensive effects of nitrate on RSA, nitrate has been termed an environmental morphogen (Guan, 2017), and substantial research has been devoted to unraveling the mechanisms through which nitrate affects RSA. The picture that has emerged is that plants employ a highly complex molecular network responsible for the sensing of internal and external nitrate status, integration of these signals, and the mounting of a suite of possible growth responses. For plants exposed to homogeneous external nitrate conditions, depending on external and hence internal nitrate levels a continuum of growth responses has been described (Giehl and Von Wirén, 2014). For very low internal nitrate status, plants engage in a survival response, repressing root growth through the CLE-CLV1 module (Araya et al., 2014). The low nitrate induced repression of the AUX/IAA ACR4/AXR5 further contributes to this survival response (Giehl et al., 2014) (Figure 1A, left). For somewhat less low internal nitrate levels plants instead display a foraging response, promoting root growth via the induction of TAR2, which results in enhanced local auxin biosynthesis (Ma et al., 2014). Additionally, this foraging response likely involves the low nitrate status induced expression of WAK4 and the downstream auxin transporter MDR4/PGP4 (Giehl et al., 2014) known to promote lateral root formation (Lally et al., 2001; Terasaka et al., 2005) (Figure 1A, second from left). Finally, for very high internal nitrate levels, a systemic repression response occurs, reducing root growth through repression of auxin sensing via the AFB3, NAC4 and OBP4 pathway (Vidal et al., 2010). Root growth may be further repressed through the HNI9 dependent repression of nitrate transport (Girin et al., 2010) (Figure 1A, right).

In addition to the above, under heterogeneous external nitrate conditions plant roots display a preferential growth of the root system in nitrate rich patches (Ruffel et al., 2011; Guan et al., 2014; Mounier et al., 2014). To investigate the mechanisms underlying this so-called preferential root foraging, split root experiments are used (Ruffel et al., 2011; Guan et al., 2014; Mounier et al., 2014) (Figures 1B,C). For sufficiently different high and low nitrate levels, a typical outcome of these experiments is that exposure to different nitrate concentrations results in substantial growth asymmetry (Figure 1B, middle, Figure 1C compare red and green lines and bars). Furthermore, growth at the high nitrate side is enhanced beyond that of a plant experiencing high nitrate at both root halves (Figures 1B,C, compare green versus light blue), whereas growth at the low nitrate side is diminished beyond that of a root system experiencing low nitrate at both sides (Figures 1B,C, compare red versus dark blue). These differences have been taken as evidence for the presence of growth promoting systemic demand signals and growth repressing systemic supply signals, respectively (Ruffel et al., 2011; Guan et al., 2014; Mounier et al., 2014).

Over the last years, several key players in the preferential foraging of roots for nitrate have been discovered. First, it was found that the dual-affinity nitrate transporter NRT1.1 acts as an auxin importer at low external nitrate levels, effectively reducing lateral root auxin levels and thus repressing lateral root growth (Krouk et al., 2010). At sufficiently high nitrate levels, NRT1.1 does not transport auxin, resulting in the absence of this repression. Instead, at higher nitrate levels NRT1.1 mediated nitrate transport enhances auxin signaling through the AFB3, NAC4, and OBP4 pathway, thereby promoting lateral root growth (Vidal et al., 2010, 2013, 2014). Additional enhancement of lateral root growth downstream of transported nitrate involves the cell wall modifying enzyme XTH9 (Xu and Cai, 2019), and enhanced auxin signaling via ANR1 (Remans et al., 2006),. Mutation of NRT1.1 severely reduces the preferential root foraging response (Remans et al., 2006; Mounier et al., 2014). A second cornerstone of preferential nitrate foraging was discovered with the elucidation of the *C*-terminally encoded peptide (CEP) demand signaling pathway. Under low external nitrate conditions, roots locally produce CEP peptides (Tabata et al., 2014), which become translocated to the shoot via the xylem. In the shoot they bind to so-called CEP receptors (CEPR) (Tabata et al., 2014), resulting in the production of CEP DOWNSTREAM 1 (CEPD1) and CEPD2 CC-type glutaredoxins (Ohkubo et al., 2017). These downstream signals travel back to the root via the phloem, upregulating the nitrate transporter NRT2.1 only in those roots perceiving sufficiently high external nitrate (Tabata et al., 2014). NRT2.1 has been shown to be an important component in nitrate dependent root growth stimulation (Little et al., 2005; Remans et al., 2006; Naz et al., 2019). While the mechanism through which NRT2.1 promotes root growth remains to be fully elucidated, in rice it has been shown that nitrate uptake leads to the production of NO, which subsequently results in elevated auxin levels via the upregulation of the auxin transporter PIN1 (Sun et al., 2018). As a consequence auxin supply to the lateral root is increased, enhancing lateral root growth. Thus, upregulation of NRT2.1 appears to also stimulate lateral root growth in the presence of nitrate through auxin, but via a different, less direct mechanism than that of NRT1.1. Finally, an important role for systemic CK signaling in preferential nitrate foraging has been recently uncovered (Ruffel et al., 2011; Poitout et al., 2018). Plant roots were found to produce CK in a nitrate dependent manner, with this CK subsequently being transported to the shoot, where it controls the expression of a large number of genes as well as impacts preferential root foraging.

Intriguingly, mutations in NRT1.1, NRT2.1, CK biosynthesis, and CK transport all strongly reduce preferential root foraging or uptake (Cerezo et al., 2001; Ruffel et al., 2011; Mounier et al., 2014). These results suggest an integrated, synergistic response network rather than mere additive actions. Still, how exactly these pathways are integrated and whether their combination is necessary and sufficient to explain preferential foraging remains unclear. First, while split root experiments are generally taken to indicate the presence of growth stimulating demand and growth suppressing supply signals, thus far no supply signal specific for heterogeneous nitrate conditions has been proposed. Additionally, while the role for systemic nitrate levels in modulating root growth dynamics was recently further substantiated (Okamoto et al., 2019), how systemic survival, foraging and repression responses are involved in generating root growth asymmetry under heterogeneous nitrate conditions has not been studied. Finally, plant organs are in a continuous competition for carbon resources, a process that may contribute to growth asymmetries but which involvement in preferential root foraging so far has not been considered.

In addition to the question which mechanisms are involved and how these are integrated to generate preferential foraging, an open question is how the extent of preferential foraging depends on the precise nitrate distribution patterns. It has been shown that preferential foraging depends on concentration differences between high and low nitrate patches, as well as their average nitrate level (Mounier et al., 2014). Larger concentration differences and lower average nitrate levels elicit a stronger preferential foraging response, which can be understood from the larger gains and need for nitrate in these situations. What is less clear is how the size or number of nitrate rich versus nitrate poor patches impacts the foraging response. As an example, if only one or a few high nitrate patches are found by the root system, excessive proliferation in those patches limits root growth elsewhere. Under these conditions, limiting the extent of preferential proliferation to maintain a minimum level of random, explorative growth for new nitrate rich patches would appear a better strategy.

Here we used a modeling approach to shed light on the above questions. To this aim we developed a first, simple model for the preferential foraging of roots in nitrate rich patches. We incrementally incorporated known regulatory mechanisms involved in adapting RSA to environmental and internal, systemic nitrate conditions into our model. Following this approach, we identified the likely involvement of systemic nitrate dependent suppression and foraging responses, as well as competition for carbon resources in preferential root foraging. Finally, we proposed a novel hypothesis for the role of long distance CK signaling in preferential root foraging suggesting it entails a nitrate supply signal modulating demand signal strength.

## Materials and Methods

### Model Equations

#### A Basic Model for Root System Growth and Internal Nitrate Dynamics

Our goal was to construct a simple root growth model enabling us to investigate the combined regulatory effects of external and internal nitrate status on root system growth and how these generate preferential foraging in nitrate rich patches. For simplicity, we described growth dynamics of (a part of) the root system in terms of changes in its cumulative length *L* (in mm). We thus ignored growth induced changes in root diameter, branching, or differences in growth dynamics between main and lateral roots. As a further simplification, we did not explicitly model shoot growth and the dependence of root growth on shoot generated photosynthesis products. Instead, we assumed that shoot leaf area is proportional to root system length, thus ignoring potential changes in root shoot ratio. Additionally, we assumed that carbon production is proportionate to shoot leaf area, ignoring potential self-shading in larger growing plants. Combined this enabled us to write the following equation for the growth dynamics in the root system:

(1) $$\frac{{\mathrm{dL}}_{x}}{\mathrm{dt}}=\frac{1}{n}\,c\,o\,n\,v\cdot r\,\sum_{x=0}^{n}L_{x}$$

where *x* is the index indicating the number of the root system compartment modeled and *n* is the total number of root compartments considered, with – unless specified otherwise – all root compartments obtaining an equal fraction 1/*n* of the total energy available for root growth. The parameter*conv* represents the conversion factor indicating the maximum rate of root length increase per unit of shoot area (assumed proportionate to overall root system length), assuming a linear dependence between shoot area and photosynthetic carbon production. Finally *r* represents the growth rate per unit length. In the basic model *r* is a constant valued parameter, in the subsequent model extensions *r* will be the product of a range of internal and external nitrate dependent growth regulatory functions. Note that this equation will result in exponential root growth dynamics, consistent with the growth dynamics observed for young *Arabidopsis thaliana* plants in absence of resource limitations, competition, or stress (Guan et al., 2014).

To model the dependence of root growth on external nitrate levels, we defined a parameter *N*_*e,x*_ per root compartment *x* that could be independently varied to simulate different environmental conditions. To model the dependence of root growth on internal plant nitrate status, we needed to define how uptake of external nitrate translates into internal plant nitrate status and signaling thereof. *In planta*, long distance, signaling of systemic plant nitrate status occurs via multiple factors, among which nitrate itself (Wang et al., 2004; Ruffel et al., 2011), nitrate metabolites (Walch-Liu et al., 2006), nitrate dependent changes in shoot-root auxin transport (Walch-Liu et al., 2006), overall plant abcissic acid (ABA) levels (Signora et al., 2002), and carbon to nitrate ratio (Malamy and Ryan, 2001). For simplicity we decided to model a single systemic nitrate pool, intended as a representation, of and signal for overall plant nitrate status We assume that uptake of external nitrate gives rise to local, internal nitrate levels (*N*_*i*_) per root system compartment, and that long distance transport of this internal nitrate gives rise to an overall, systemic nitrate level *N*_*s*_. Internal local nitrate levels thus increase through uptake of external nitrate (*N*_*e*_) from the environment and lose nitrate through transporting it to the system level nitrate pool. This nitrate pool in turn loses nitrate to turnover and maintenance of plant tissue as well as exudation. To take into account the uptake of external nitrate by both high and low affinity transporters (Crawford and Glass, 1998), we considered both saturated and non-saturated nitrate uptake. Combined this led to the following equations:

(2) $$\frac{{\mathrm{dN}}_{i,x}}{\mathrm{dt}}=\left({\text{up}}_{1}\,\frac{N_{e,x}}{N_{e,x}+{\text{K}}_{\text{up}}}+{\text{up}}_{2}\,N_{e}\right)\,L_{x}-T_{u\,p}\,N_{i,x}$$

(3) $$\frac{{\mathrm{dN}}_{s}}{\mathrm{dt}}={\text{T}}_{u\,p}\,\sum_{x=0}^{n}N_{i},x-u_{m}\,\sum_{x=0}^{n}L_{x}-{\text{eN}}_{s}$$

where *up*_*1*_ is the maximum uptake rate of the high affinity transporters, *K*_*up*_ is the concentration at which these high affinity transporter operate at half maximum velocity, *up*_*2*_is the uptake rate of low affinity transporters that for simplicity are assumed not to saturate, *T*_*up*_ is the rate of transport of nitrate from the local to the systemic nitrate pool, *u*_*m*_ is the rate of nitrate loss to tissue maintenance and turnover and *e* is the rate of nitrate loss to exudation. Note that dynamic, root foraging induced changes in external nitrate levels were ignored in our model. Values and units of the parameters used in Eqs 1–3 are given in Table 1. Parameter values and units for subsequent model equations are given in Table 2.

**TABLE 1:** Units and values of model parameters.

**Model parameters**	**Units**	**Values**
*up*_*1*_	*micromole*⋅*mm*^−1^⋅*h*^−1^	0.6
*K*_*up*_	*micromole*⋅*L*^−1^	75
*up*_*2*_	*L*⋅*mm*^−1^⋅*h*^−1^	0.000006
*N*_*e*_	*micromole*⋅*L*^−1^	110–11,000
*T*_*up*_	*h*^−1^	3.8
*u*_*m*_	*micromole*⋅*mm*^−1^⋅*h*^−1^	0.1
*e*	*h*^−1^	1.5
*conv*	dimensionless	0.01

**TABLE 2:** Parameter settings for model extensions.

**Model parameters**	**Units**	**Values**
**Local Ne signaling**		
*a*_local_	dimensionless	0.35
*K*_local_	*micromole*⋅*mm*^−1^	200
**CEP demand signaling**		
*p*_CEP_	*micromole*⋅*mm*^−1^⋅*h*^−1^	0.1
*K*_CEP_	*micromole*⋅*mm*^−1^	250
*T*_CEP_	*h*^−1^	0.1
*d*_CEP_	*h*^−1^	0.001
*a*_CEP_	Dimensionless	0.5
*K*_NRT2.1,CEP_	*micromole*⋅*mm*^−1^	1
*K*_CEP,NE_	*micromole*⋅*L*^−1^	750
**CK supply signaling**		
*p*_CK_	*micromole*⋅*mm*^−1^⋅*h*^−1^	0.1
*K*_CK_	*micromole*⋅*mm*^−1^	750
*T*_CK_	*h*^−1^	0.1
*d*_CK_	*h*^−1^	0.001
*a*_CK_	Dimensionless	0.1
*K*_CEP,CK_	*micromole*⋅*mm*^−1^	2
**Systemic N survival signaling**		
*a*_basic_	*h*^−1^	0.5
*k*_basic_	*micromole*⋅*mm*^−1^	0.04
**Systemic N foraging signaling**		
*a*_systfor_	dimensionless	1
*K*_systfor_	*micromole*⋅*mm*^−1^	0.12
**Systemic N repression signaling**		
*K*_systrepr_	*micromole*⋅*mm*^−1^	0.4

#### Single Root, Split Root, and Patch Experiments

To investigate the effect of changes in homogeneous external nitrate concentration on root system growth, we applied a single root compartment (*n=1*). To simulate split root experiments in which different root halves (potentially) experience different external nitrate levels, we applied two root compartments (*n=2*). Finally, to investigate the role of isolated nitrate patches on root system growth, we simulated a large number (in this study *n* = 16) of root system compartments, with only one of these containing high nitrate levels. Importantly, without additional growth regulation following these equations differences in the external nitrate levels will result in differences internal and systemic nitrate levels, but not root growth (Eq. 1).

### Dimensions and Parametrization of the Model

#### Units of Model Variables

Note that since in our simplified model we worked only with length, and not radius, volume or weight of the root system, for convenience concentrations were computed per unit length. Thus, *L*, length of (part of) the root system is in mm, *N*_*i*_, internal nitrate amount, and *N*_*s*_, systemic nitrate signaling amount, are in micromole, whereas $\left[N_{i}\right]=\frac{N_{i}}{L}$ internal nitrate concentration, and $\left[N_{s}\right]=\frac{N_{s}}{L}$ systemic nitrate signaling concentration, are in micromole/mm.

#### Parameter Values

For the maximum uptake rate of high affinity transporters, depending on the study and the specific nitrate transporter studied, values between 0.3 and 8 μmol/g freshweight/h are reported (Crawford and Glass, 1998). Since we only incorporated a single, generalized high affinity nitrate uptake transporter in our baseline model, we chose an intermediate value of 3.6 μmol/g freshweight/h. Next, since in our simplified model all is per unit length, we needed to convert this value to μmol/mm/h. For this we used data from a study on *Brachypodium* (Sasse et al., 2019), reporting an approximately 1.2:1 ratio between 1 g freshweight and 1 cm root length, resulting in a rounded off 0.6 μmol/mm/h value for *up*_*1*_. Similarly, for *K*_*up*_, also depending on study and nitrate transporter studied, values ranging from 6 to 100 μmol/L have been reported (Crawford and Glass, 1998). Again we took an intermediate value of 50 μmol/L.

**FIGURE 2:**
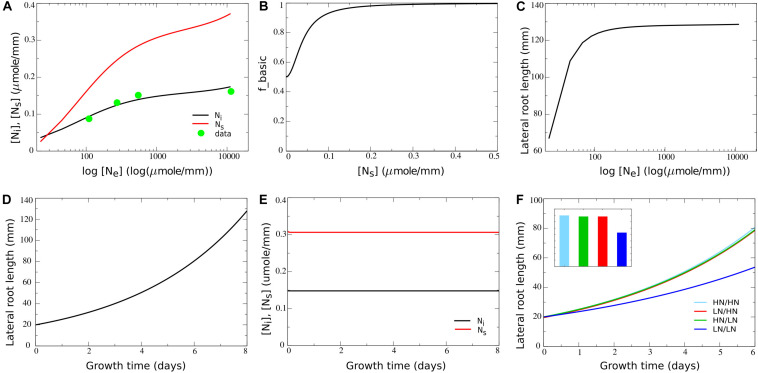
Incorporating a saturating dependence on systemic nitrate levels. **(A)** Internal and systemic nitrate levels as a function of external nitrate for the basic model settings in which growth is nitrate independent. For comparison purposes, data obtained by Gruber et al. (2013) are added after conversion of these data to the same dimensions as our model (for details see section “Materials and Methods”). **(B)** Model survival response: growth rate decrease as a function of systemic nitrate levels. **(C)** Cumulative root system length after 8 days of growth (in mm) as a function of external nitrate levels in the model including the survival response. **(D)** Exponential root growth dynamics for an external nitrate level of 1,000 μM. **(E)** Internal and systemic nitrate dynamics for the root growth shown in **(D)**. **(F)** Growth dynamics (main figure) and final size after 6 days (inset) of single root halves in split root experiments. LN/LN:(dark blue): low nitrate levels experiencing root system half for which the other root system half is also experiencing low nitrate levels; LN/HN (red): low nitrate root system half for which the other root system half is experiencing high nitrate; HN/LN (green): high nitrate root system half for which the other half is experiencing low nitrate; HN/HN (light blue): high nitrate root system half for which the other half is also experiencing high nitrate. See also Figures 1B,C.

Given the simplified nature of our model, for other parameters values could not be directly derived from available data. For example, *T*_*up*_, the rate of transport of internal nitrate to the systemic nitrate pool, is in fact a compendium of the characteristics of nitrate transporters in phloem and xylem, as well as their numbers and distribution, the distance covered, etc. Therefore, instead we fitted *T*_*up*_ and *up*_*2*_ to reproduce the experimentally observed dependence of internal nitrate concentrations on external nitrate concentrations. For this we used data from a study by Gruber et al. (2013), in which *Arabidopsis* plants were exposed to a range of external nitrate concentrations ranging from 110 to 11,000 μmol/L. Internal nitrate concentrations reported in Gruber et al. (2013) are shoot/root levels of 72/48; 66/45, 48/39, and 29/na mg/g dryweight for externally applied nitrate of 11,400, 550, 275, and 110 μmol/L, respectively. First, the not available (na) root internal nitrate level for 110 μmol/L external nitrate was extrapolated from the available root and shoot values. Assuming that for 110 μmol/L the ratio between shoot and root nitrate levels is 1.1, this resulted in a value of 26 mg/g dryweight. Thus, shoot nitrate levels vary 2.5-fold, and root nitrate levels vary 1.8-fold over a 100-fold change in external nitrate levels. Next, we converted the internal nitrate levels to micromole/g freshweight. For this we assumed a fourfold weight difference between fresh and dry weight (effectively assuming 75% of plant mass consists of water), consistent with classical experimental values (van de Sande-Bakhuyzen, 1928). Additionally, we used the molecular weight for nitrate of 62,0049 g/mol. This resulted in converted root internal nitrate levels of 0.194, 0.181, 0.157, and 0.105 μmol/g freshweight. Finally, since we described root growth in terms of length increase, we converted these internal nitrate levels to micromole/mm. For this we again used data from a study on *Brachypodium* and derived a 1.2:1 ratio between 1 g freshweight and 1 cm root length (Sasse et al., 2019). After this final conversion we obtained root internal nitrate levels of 0.161, 0.151, 0.131, and 0.087 for 11,400, 550, 275, and 110 μmol/L external nitrate, respectively. By taking *T*_*up*_ = 3.8*h*^−1^ and *up*_2_ = 0.000006*L*⋅*mm*^−1^⋅*h*^−1^ we obtained a good fit for the dependence of internal nitrate levels on external nitrate between the model and the experimental data (see Figure 2A in section “Results,” compare black line with green circles).

Parameter values for *u*_*m*_ (0.1*mmol*⋅*mm*^−1^⋅*h*^−1^) and *e* (1.5*h*^−1^) were chosen such that systemic nitrate levels show a larger range of variation as a function of external nitrate compared to the local internal nitrate levels (Figure 2A, compare black and red lines). The reason for doing this is that systemic nitrate level is known to affect root system growth in various ways, at different systemic nitrate levels. A survival response, during which root growth is strongly repressed occurs for very low systemic nitrate levels. In contrast, for somewhat higher systemic nitrate levels a foraging response promoting root growth is induced. Finally, for very high systemic nitrate levels, systemic repression reduces root growth (Giehl and Von Wirén, 2014). In order to incorporate these different effects into our model in a robust manner, we should be able to activate these effects at sufficiently different systemic nitrate concentrations, requiring a large enough range of systemic nitrate levels to occur in our model.

As a final parameter we needed to determine the value for *conv*. For this, we made use of the fact that we can write an analytical solution for Eq. 1:

(4) $$L\,\left(t\right)=L\,\left(0\right)\,e^{c\,o\,n\,v\cdot r\cdot t}$$

Typically, in split root experiments, split root conditions are started when the first order laterals have grown to a size of 2–4 cm (see Guan et al., 2014). We therefore took the start size of a single side of the root system (with only one side present in case of an unsplit root system) as *L*(0) = 20*mm*. Experimental data also indicate that the maximum cumulative length of secondary laterals in one root half after 10 days of treatment lies around 24 cm, and far exceeds the primary lateral root length (Guan et al., 2014). Thus, we assumed that after 10 days the maximum size of half the root system is 36 cm (1.5 times 24 cm) and hence the maximum size for the whole root system size lies around 72 cm, so *L*(240*h*) = 720*mm*. Since this is the maximum root system size occurring under favorable conditions, i.e., conditions promoting growth, we assumed that *r* = 1.5⋅*h*^−1^ rather than being equal to 1. Together this resulted in:

$$720\,\mathrm{mm}=20\,{\mathrm{mme}}^{c\,o\,n\,v\cdot 1.5\,h^{-1}\cdot 240\,h}$$

from which we solved *conv* = 0.01.

Combined this resulted in the parameter settings shown in Table 1.

### Model Extensions

#### Local and Systemic CEP Dynamics

To model the effect of nitrate demand signaling on preferential root foraging (see section “Results”), we extended our model with a local nitrate dependent, decreasing, non-linear production of CEP. Locally produced CEP is subsequently transported to a systemic CEP pool, where it undergoes degradation. To describe these dynamics, we extended our model with the following equations:

(5) $$\frac{{\mathrm{dCEP}}_{x}}{\mathrm{dt}}={\text{p}}_{\text{CEP}}\,\frac{K_{\text{CEP}}^{2}}{K_{\text{CEP}}^{2}+N_{E,x}^{2}}\,L_{x}-T_{\text{CEP}}\,{\text{CEP}}_{x}$$

(6) $$\frac{{\mathrm{dCEP}}_{s}}{\mathrm{dt}}=T_{\text{CEP}}\,\sum_{x=0}^{n}{\text{CEP}}_{x}-d_{\text{CEP}}\,{\text{CEP}}_{S}$$

where *p*_CEP_ is the maximum rate of CEP production rate, *K*_CEP_ is the external nitrate concentration at which CEP production reaches its half maximum rate, *T*_CEP_ is the rate of transport from the local to systemic CEP pool, and *d*_CEP_ is the degradation rate of CEP. Note that, similar as for nitrate, *CEP*_*x*_ and *CEP*_*s*_ represent the amounts of locally produced and systemic CEP, while $\left[C\,E\,P_{x}\right]=\frac{C\,E\,P_{x}}{L_{x}}$ and $\left[C\,E\,P_{s}\right]=\frac{C\,E\,P_{s}}{\sum_{x}L_{x}}$ represent the concentrations of local and systemic CEP. For parameter values and dimensions, see Table 2.

#### Local and Systemic CK Dynamics

To incorporate the effect of nitrate supply signaling on preferential root foraging (see section “Results”), we added to our model CK dynamics. CK is produced locally, in an external nitrate dependent manner, and transported to a systemic CK pool, where it undergoes degradation. CK dynamics were modeled using the following equations:

(7) $$\frac{{\mathrm{dCK}}_{x}}{\mathrm{dt}}={\text{p}}_{\text{CK}}\,\frac{N_{E,x}^{2}}{K_{\text{CK}}^{2}+N_{E,x}^{2}}\,L_{x}-T_{\text{CK}}\,{\text{CK}}_{x}$$

(8) $$\frac{{\mathrm{dCK}}_{s}}{\mathrm{dt}}={\text{T}}_{\text{CK}}\,\sum_{x=0}^{n}{\text{CK}}_{x}-d_{\text{CK}}\,{\text{CK}}_{S}$$

where *p*_CK_ is the maximum rate of CK production, *K*_CK_ is the external nitrate concentration at which CK production reaches its half maximum rate, *T*_CK_ is the rate of transport from the local to systemic CK pool, and *d*_CK_ is the degradation rate of CK. Again, similar as for nitrate and CEP, *CK*_*x*_, and *CK*_*s*_ represent the amounts of locally produced and systemic CK, while $\left[C\,E\,P_{x}\right]=\frac{C\,E\,P_{x}}{L_{x}}$ and $\left[C\,K_{s}\right]=\frac{C\,K_{s}}{\sum_{x}L_{x}}$ represent the concentrations of local and systemic CK. For parameter values and dimensions, see Table 2.

#### Parameter Settings for Model Extensions

In the section “Results,” as well as above, we described how our baseline model is extended to incorporate the various known aspects of external and internal nitrate status dependent growth regulation. Parameters, values and dimensions involved in these model extensions are listed in Table 2.

#### Model Code

Model code was written in C++, and is freely available as open source code.^1^ Model output was visualized using the Xmgrace graph plotting tool.

## Results

### Establishing a Baseline Root Growth Model

To establish a baseline model for *Arabidopsis thaliana* root growth in which subsequent extensions can be built, we started with a single, non-split root system. This root system takes up external nitrate from the environment, transports this nitrate into a systemic nitrate pool, and grows (see section “Materials and Methods,” Eqs 1–3).

In nature, soil nitrate levels have been found to vary five to sevenfold with depth in the soil (Angle et al., 1993; Jin et al., 2015), three to fivefold with seasonal changes (Taylor et al., 1982; Weil and Brady, 1996; Hellebrand et al., 2005), and threefold even between similar soils (Jin et al., 2015). Combined, this suggests that plant roots experience variations in soil nitrate levels of up to two orders of magnitude. At the same time experimental data show that 100-fold changes in external nitrate result in only 1.82- to 2.5-fold changes in leaf and root nitrate levels (Gruber et al., 2013). To be able to simulate this we fitted model parameters to experimental data (see section “Materials and Methods”), initially assuming no nitrate dependent growth regulation (*r* = 1*h*^−1^). Figure 2A plots simulated local internal and systemic nitrate levels as a function of external nitrate and shows a good agreement between simulated and experimentally obtained internal nitrate levels.

Next, we introduced the first, basic dependence of root growth on nitrate levels. Obviously, plant growth ultimately depends on the carbon generated through photosynthesis. Photosynthesis in turn is highly dependent on the protein Rubisco and as such also dependent on nitrate levels. Additionally, plants have been shown to display a survival response for low external (and hence systemic) nitrate levels, repressing root growth via the CLE-CLV1 module (Araya et al., 2014) as well as through ACR4/AXR5 (Giehl et al., 2014). Based on this we incorporated a saturating dependence of growth rate on systemic nitrate levels writing:

(9a) $$r=f_{\text{basic}}$$

with

(9b) $$f_{\text{basic}}=a_{\text{basic}}+\left(1-a_{\text{basic}}\right)\,\frac{{\left[N_{s}\right]}^{2}}{{\left[N_{s}\right]}^{2}+{\text{K}}_{\text{basic}}^{2}}$$

where *a*_basic_ is the [*N*_*s*_] independent and (1−*a*_basic_) the [*N*_*s*_] dependent fraction of *f*_basic_, and *K*_basic_ is the systemic nitrate level at which the systemic nitrate dependent fraction of the growth rate is half maximal. Based on the systemic nitrate levels occurring in Figure 2A, to ensure that survival responses occur only at very low nitrate levels, we choose *K*_basic_ = 0.04*micromol*⋅*mm*^−1^. Next we needed to decide on the value for *a*_basic_. In split root experiments in which both root halves are exposed to low or even absent nitrate, some root growth still occurs (Ruffel et al., 2011; Guan et al., 2014; Mounier et al., 2014). This likely results from the exposure to external nitrate prior to the start of the split root experiments causing the presence of stored nitrate. Since we did not include nitrate stores in our simple model, yet aimed to simulate split root experiments, we modeled this limited reduction of root growth by the survival response by using a value of *a*_basic_ = 0.5*h*^−1^. Combined this resulted in the dependence of *f*_basic_ on *N*_*s*_ as shown in Figure 2B. Figure 2C shows root system length after 8 days of growth as a function of external nitrate levels based on these growth rate settings. We observed a saturating effect of external nitrate on overall root system size, yet without root growth being fully abolished at very low external nitrate levels. When plotting the temporal dynamics of root growth at a single external nitrate level (*N*_*e*_ = 1000*micromoleL*^−1^), we obtained an exponential increase in root growth length over time (Figure 2D), as has been observed experimentally (Guan et al., 2014). Additionally, we observed that after an initial transient, steady state local and systemic nitrate concentrations are reached despite continued growth (Figure 2E).

Next we investigated root growth dynamics produced when simulating classical split root experiments, in which root halves are exposed to either very low (25 μM) or high (5,000 μM) external nitrate levels. Note that for clarity, for the situations in which both root halves are exposed to the same nitrate level only the length of a single root half was shown. It can be seen that root growth was less when both root halves were exposed to very low nitrate levels, yet did not differ much between the situation when only one or both root halves were exposed to a high nitrate level (Figure 2F). This is consistent with the applied saturating dependence of root growth on systemic nitrate levels. Additionally, we observed no differences between left and right root halves of plants experiencing heterogeneous external nitrate conditions. This logically follows from the fact that growth in the current model settings only depended on systemic but not local internal nitrate levels.

In the next sections, we will incrementally add additional, nitrate dependent regulatory effects on root growth to investigate how these may help explain the preferential nitrate foraging root phenotype. Importantly, beyond the point of introducing a particular regulatory function, all subsequently discussed model variants will include that regulatory function. Practically this implies that the effective growth rate *r* will become the product of an increasing number of growth regulatory functions *f*.

### Local Nitrate Signaling

Local nitrate levels have been shown to affect lateral root growth root. One of the key players involved in this local response is the nitrate transceptor NRT1.1. For low external nitrate levels, NRT1.1 has been shown to function as an auxin importer, resulting in the reduction of local auxin levels and thereby inhibiting lateral root growth. In contrast, for higher external nitrate levels, NRT1.1 does not transport auxin, therefore, not having this negative effect on root growth (Krouk et al., 2010). Additionally, for higher external nitrate levels NRT1.1 positively influences auxin signaling and hence lateral root growth via the AFB3, NAC4, OBP4 pathway (Vidal et al., 2010, 2013, 2014), as well as ANR1 (Remans et al., 2006).

To investigate the contribution of local nitrate sensing to preferential foraging with our model we extended our baseline model by incorporating a root growth promoting function. This function emulates the above described effects on auxin transport and signaling and depends in a saturating manner on the local external nitrate level:

(10a) $$r=f_{\text{basic}}*f_{\text{local}},\text{with}$$

(10b) $$f_{\text{local}}=a_{\text{local}}\,\frac{N_{e}^{2}}{N_{e}^{2}+K_{\text{local}}^{2}}+\left(1-a_{\text{local}}\right)$$

where *a*_local_ represents the *N*_*e*_ dependent and (1−*a*_local_) the *N*_*e*_ independent fraction of *f*_local_, and *K*_local_ represents the external nitrate concentration at which the external nitrate dependent fraction reaches half of its maximum value.

**FIGURE 3:**
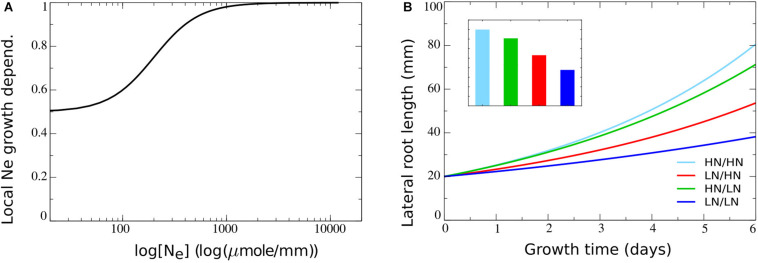
Including the dependence of root growth on local nitrate levels. **(A)** Model local stimulation response: growth rate dependence on local external nitrate levels. **(B)** Growth dynamics (main figure) and final size after 6 days (inset) of single root halves in split root experiments. Colors same as in Figures 1B,C and 2F.

Parameter values were chosen such (see Table 2) that a baseline growth rate of 0.65 arises if no external nitrate is present, with growth rates increasing to one as external nitrate increases (Figure 3A). As a consequence, relative to an external nitrate level of say 250 μM which resulted in a growth rate of approximately 0.8, both external nitrate dependent decreases and increases in growth rate may occur, consistent with the above described experimental data. In Figure 3B, outcomes of split root simulations under these new model settings are shown. We observed that with the impact of external nitrate levels added, root length differences between plants experiencing only very low or only very high nitrate concentrations increased (Figure 3B, dark blue versus light blue). Furthermore, as expected, we now saw an asymmetry in root lengths in plants experiencing heterogeneous external nitrate concentrations, with the root half experiencing higher nitrate levels growing longer. However, root system length on the high nitrate side was lower compared to the situation in which both root halves experienced high nitrate levels. On a similar note, root system length on the low nitrate side was higher as compared to the situation in which both root halves experienced low nitrate levels. This is the reverse of what is observed experimentally (Figure 3B). This can be easily understood from the dependence of root growth on both *r*, and hence local nitrate, as well as on overall root system size *L* (assumed proportional to shoot size and hence carbon availability for growth) in our model. In case of the heterogeneous split root system, local nitrate stimulates root growth in one of the two root halves. This results in an *L* and hence root growth rate intermediate to that of plants experiencing low nitrate at both sides and plants experiencing high nitrate at both sides.

### Systemic Demand Signaling

**FIGURE 4:**
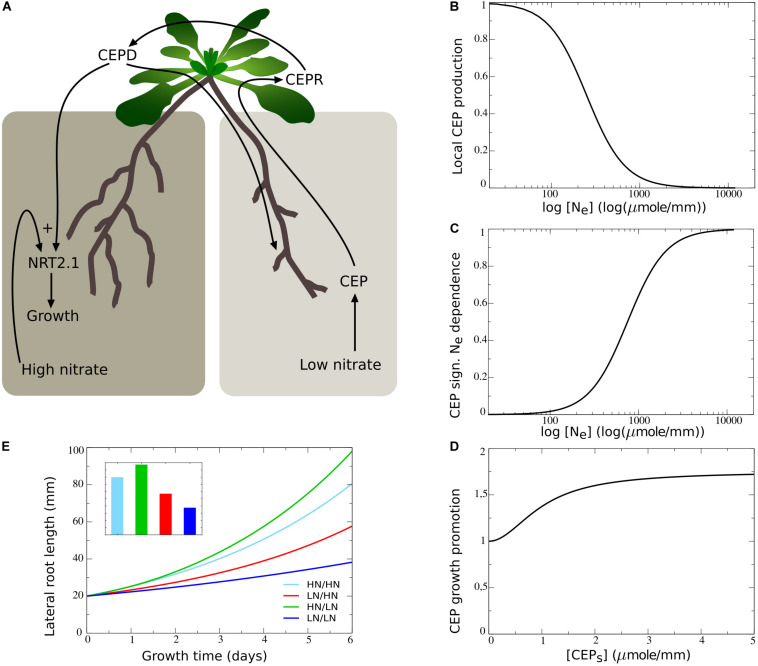
Including the dependence of root growth on CEP-mediated demand signaling. **(A)** Schematic depiction of the CEP-mediated demand signaling system, showing the low nitrate induced production of CEP (1), the CEPD1/CEPD2 and local nitrate dependent upregulation of NRT2.1 (2), and the NRT2.1 dependent stimulation of root growth (3). **(B)** Dependence of the rate of CEP production on the low nitrate side on local external nitrate levels. **(C)** Modulation of CEP signaling effect on root growth promotion on the high nitrate side **(D)** on local external nitrate. **(D)** Dependence of root growth promotion on the high nitrate side on systemic CEP signaling levels. **(E)** Growth dynamics (main figure) and final size after 6 days (inset) of single root halves in split root experiments. Colors the same as in Figures 1B,C and 2F.

In contrast to the results obtained above, plants show a preferential increase in lateral root lengths at the high nitrate side as compared to plants experiencing high nitrate at both sides (Figure 1B). This suggests the involvement of a growth promoting systemic demand signal. Recently, at least part of such a systemic nitrate lack signaling system has been uncovered. It was shown that under low external nitrate levels lateral roots produce CEP peptides, which in the shoot bind to CEPR and cause the production of CEPD1 and CEPD2 downstream signals that travel back to the root. CEP signaling combined with the local presence of sufficient nitrate subsequently leads to the upregulation of NRT2.1 (Tabata et al., 2014; Ohkubo et al., 2017), and results in upregulation of nitrate uptake as well as nitrate dependent root growth (Little et al., 2005; Remans et al., 2006; Naz et al., 2019) (Figure 4A).

To incorporate this mechanism in our model we added the local nitrate dependent production of CEP, with CEP production decreasing in a non-linear saturating manner with increasing external nitrate levels (Figure 4B). Additionally, we modeled the transport of CEP to the shoot, where it enters the systemic CEP pool and has a certain rate of turnover (see section “Materials and Methods,” Eqs 5, 6).

To restrict the number of variables included in our model, rather than explicitly modeling CEPR and downstream signals, we incorporated a direct dependence of root growth on systemic CEP signaling (*CEP*_*S*_). Our CEP-dependent growth function was chosen such that in absence of systemic CEP and other regulations, a baseline growth rate of one occurred, while in presence of systemic CEP growth was enhanced (Figure 4D):

$$\text{r}={\text{f}}_{\text{basic}}*f_{\text{local}}*f_{\text{CEP}}\,\text{with}$$

(11a) $$f_{\text{CEP}}=1+a_{\text{CEP}}\,\frac{{\left[{\text{CEP}}_{s}\right]}^{2}}{{\left[{\text{CEP}}_{s}\right]}^{2}+K_{\mathrm{NRT2}\,.1,\mathrm{CEP}}^{2}}*g_{N_{E}}\,\text{with}$$

(11b) $$g_{N_{E}}=\frac{N_{e,x}^{2}}{N_{e,x}^{2}+K_{\mathrm{CEP},N_{e}}^{2}}$$

where *a*_CEP_ is the maximum CEP signaling induced increase in growth rate, *K*_NRT2.1,CEP_ is the CEP level at which this growth rate increase reaches it’s half maximum value, and *K*_CEP,N_E_ is the local nitrate level at which this growth rate increase reaches it’s half maximum value. By multiplying the CEP dependent part of this function with *g*_*Ne*_, which depends in a saturating manner on local external nitrate (Figure 4C) we incorporated that CEP mediated growth promotion only occurs in the presence of local nitrate. Thus, local production of CEP is inversely proportional to local external nitrate levels (Eq. 4), whereas local growth promotion by systemic CEP signaling requires presence of sufficient local external nitrate (Eq. 10). Combined this should cause nitrate lack in one location, via local production of CEP, to induce growth promotion in locations without a lack of nitrate, consistent with experimental observations.

Figure 4E shows how combining this mechanism with the earlier incorporated growth regulating mechanisms resulted in the preferential enhancement of root growth at the high nitrate side.

### Systemic Repression

In addition to the high nitrate side having longer lateral roots in heterogeneous as compared to homogeneous conditions, root length under homogeneous high external nitrate conditions has been observed to be very low (Figure 1B). This reduction of overall lateral root length under high nitrate has been attributed to systemic repression (Figure 1A) (Giehl and Von Wirén, 2014). This reduced investment in root system growth under conditions of superfluous nutrient availability is considered an adaptive response. Systemic repression is mediated through the repression of auxin sensing via the AFB3, NAC4, OBP4 pathway (Vidal et al., 2010), and may also involve the HNI9 mediated repression of nitrate transport (Girin et al., 2010).

**FIGURE 5:**
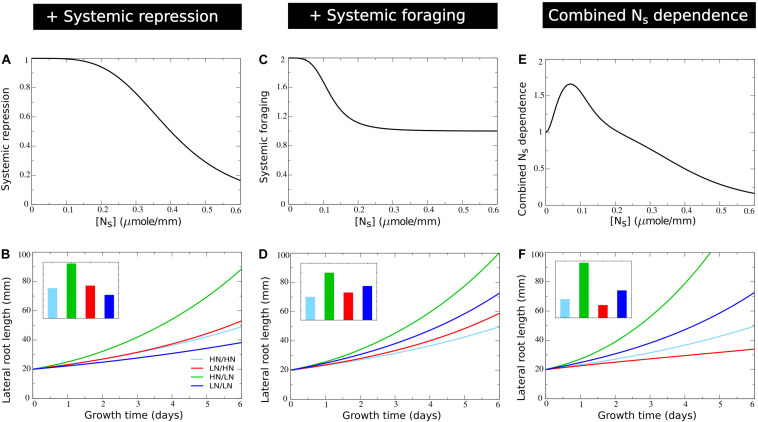
Incorporating systemic repression, systemic foraging and competition for carbon. **(A)** Model systemic repression response: growth rate decrease as a function of increasing systemic nitrate levels. **(B)** Growth dynamics (main figure) and final size (inset) of individual root halves in split root experiments when a systemic repression response is added. **(C)** Model systemic foraging response: growth rate increase as a function of decreasing systemic nitrate levels. **(D)** Growth dynamics (main figure) and final size (inset) of individual root halves in split root experiments when a systemic root foraging response is added. **(E)** Growth rate modulation occurring as a combination systemic survival, foraging, and repression responses (shown is *f*_basic_*f_systrepr_*f_systfor_). **(F)** Growth dynamics (main figure) and final size (inset) of individual root halves in split root experiments when competition for carbon is added. For **(B)**, **(D)**, and **(F)** colors are same as in Figures 1B,C and 2E.

To further improve the realism of our model we therefore incorporated a function describing the decrease of root growth rate with systemic nitrate levels:

(12) $$f_{\text{systrepr}}=\frac{K_{\text{systrepr}}^{4}}{K_{\text{systrepr}}^{4}+{\left[N_{s}\right]}^{4}}$$

where *K*_systrepr_ is the systemic nitrate concentration at which *f*_systrepr_ has decreased to half its maximum value. Based on the range of systemic nitrate levels observed in Figure 2A we choose *K*_systrepr_ = 0.4*micromole*⋅*mm*^−1^, ensuring repression only occurs for very high internal nitrate levels (Figure 5A). As expected, when comparing Figure 5B to Figure 4E mostly the root lengths of the plant experiencing high nitrate levels on both sides have decreased.

### Systemic Foraging

Thus far, our model offers an explanation for only one half of the preferential root foraging phenotype. *In planta*, in addition to a preferential increase in lateral root lengths on the high nitrate side, also a preferential decrease on the low nitrate side is observed. That is, lateral root length on the low nitrate side is lower than the lateral root lengths of plants experiencing low nitrate levels on both sides. In our current model settings, this was difficult to reproduce due to the very low root lengths occurring for plants experiencing homogeneous low nitrate levels. However, in plants, in between the extremely low systemic nitrate levels inducing a survival response and the very high nitrate levels inducing systemic repression, a third growth response occurs. This response is referred to as a root foraging response, and occurs for moderately low systemic nitrate levels (Giehl and Von Wirén, 2014). Since the foraging response promotes growth at low systemic nitrate levels, yet does not repress growth at high nitrate levels, our function was chosen such that for higher systemic nitrate level a baseline growth rate of one occurs, while in presence of low systemic nitrate levels this growth rate was enhanced (Figure 5C). Again, we incorporated this growth affecting mechanism incrementally to our model using the following equation:

(13) $$f_{\text{systfor}}=1+a_{\text{systfor}}\,\frac{K_{\text{systfor}}^{4}}{{\left[N_{s}\right]}^{4}+K_{\text{systfor}}^{4}}$$

with *a*_systfor_ the amplitude with which low *N*_*S*_ stimulates growth, which becomes half maximal at an *N*_*S*_ concentration of *K*_systfor_. We choose *K*_systfor_ = 0.12*micromole*⋅*mm*^−1^, ensuring it to occur for higher *N*_*S*_ levels than the survival response (*K*_basic_ = 0.04*micromole*⋅*mm*^−1^) and for lower *N*_*S*_ levels than the systemic repression response (*K*_systrepr_ = 0.4*micromole*⋅*mm*^−1^) (Figure 5C). In Figure 5E the combined effect of systemic nitrate on root growth rate, incorporating, survival, foraging, and systemic repression responses is shown. Incorporating our foraging response led to an elevation of root growth of plants experiencing homogeneous low nitrate. This finally resulted in a lower root length at the low nitrate side of plants experiencing heterogeneous nitrate levels as compared to plants experiencing homogeneous low nitrate levels (Figure 5D).

### Carbon Allocation

While we had meanwhile incorporated all major known local and systemic nitrate effects on root growth we still observed that root length at the low side of a plant experiencing heterogeneous nitrate levels was longer than in a plant experiencing homogeneous high nitrate conditions (Figure 5D). This contrasts with available experimental data (Figure 1B) (Ruffel et al., 2011; Guan et al., 2014; Mounier et al., 2014). It is of course likely that the simplified nature of our model limits its abilities to reproduce all aspects of plant root nitrate foraging, and that certain important regulatory mechanisms remain to be discovered. Still, we reasoned that an important known growth regulatory aspect was missing in our model, namely carbon allocation. Besides being regulated by hormones, microRNAs, peptides and gene expression, plant organ growth foremost depends on available carbon. Thus far, we assumed both root halves to receive equal amounts of the carbon available for the root system (see section “Materials and Methods,” Eq. 1). However, it is well known that the allocation of carbon to organs strongly depends on their sink strength and hence potential growth rate (Marcelis, 1996). Additionally, a correlation between nitrate presence, root growth and carbon allocation has been reported in soybean (Fujikake et al., 2003). To incorporate this aspect into our model we modified Eq. 1 into:

(14a) $$\frac{{\mathrm{dL}}_{x}}{\mathrm{dt}}={\text{g}}_{\text{carbon}}\,c\,o\,n\,v\cdot r_{x}\,\sum_{x=0}^{n}L_{x}\,\text{with}$$

(14b) $$g_{\text{carbon}}=\frac{r_{x}\,\frac{L_{x}}{\sum_{x=0}^{n}L_{x}}}{\sum_{x=0}^{n}r_{x}\,\frac{L_{x}}{\sum_{x=0}^{n}L_{x}}}$$

Put simply, the $\frac{1}{n}$ fraction in Eq. 1 that represented all *n* root compartments obtaining an equal fraction of carbon resources was replaced by a factor *g*_carbon_ describing carbon allocation as a function of relative growth rate (which would simply be $\frac{r_{x}}{\sum_{x=0}^{n}r_{x}}$), yet with the growth rates of the compartments weighted based on their relative size $\left(\frac{L_{x}}{\sum_{x=0}^{n}L_{x}}\right)$. As an example, if we assume *n=2* after some rewriting one would obtain for compartment *x=1* $g_{\text{carbon}}=\frac{r_{1}\,L_{1}}{r_{1}\,L_{1}+r_{2}\,L_{2}}$.

Figure 5F shows how incorporating competition for carbon amplified differences in root length between the high and low nitrate side of plants. This finally resulted in the low nitrate side now also having shorter root lengths than a plant exposed on two sides to high nitrate.

### Role of Systemic CK Signaling

**FIGURE 6:**
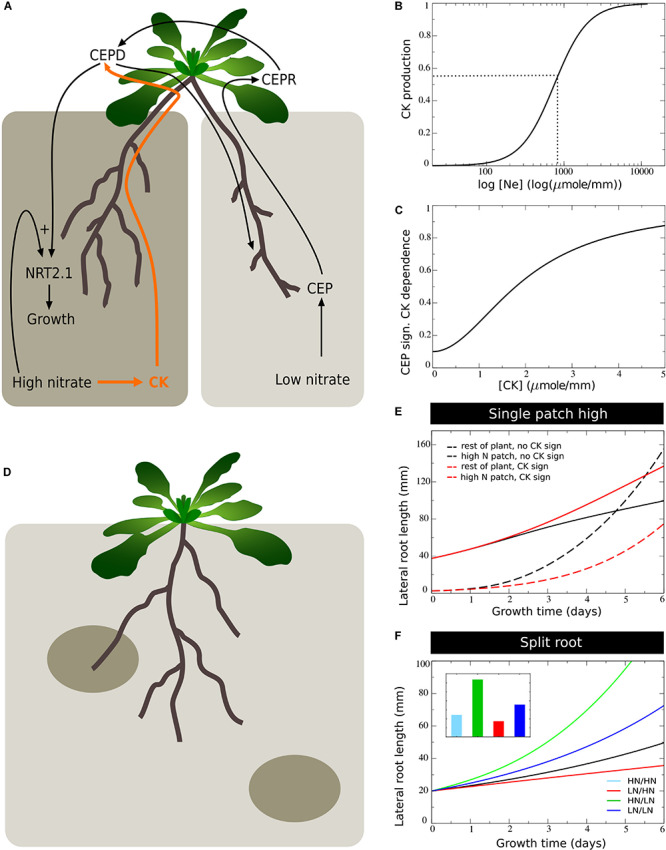
Incorporating systemic supply dependence of systemic demand signaling. **(A)** Schematic depiction of the proposed modulation of CEP demand signaling by CK supply signaling. **(B)** Production of CK as a function of external nitrate levels. **(C)** CK dependent modulation of CEP signaling strength. **(D)** Plant growing in a patchy nitrate environment, with part of its root system in a high nitrate patch and another high nitrate patch remaining to be discovered. **(E)** Growth dynamics in single compartment experiencing high nitrate and cumulative growth dynamics in other low nitrate experiencing compartments when CK supply signaling is not (black lines) or is (red lines) modulating CEP demand signaling strength. **(F)** Growth dynamics (main figure) and final size (inset) of individual root halves in split root experiments when CK supply signaling modulates CEP demand signaling. Colors same as in Figures 1B,C and 2E.

It has been recently demonstrated that systemic signaling occurring via root produced cytokinins also plays an important role in preferential root foraging. Based on the observation that triple CK biosynthesis mutants show hardly an increase in LR length on the high nitrate side compared to homogeneous nitrate conditions, this CK based signaling system was interpreted as a demand signal (Poitout et al., 2018; Ruffel et al., 2011). Still, root CK production correlates with local root nitrate levels, suggesting CK to rather be a supply signal (Takei et al., 2002). This implies that for the demand driven upregulation of lateral root growth to function not only local nitrate presence signaling is required but also systemic supply signaling. Consistent with this interpretation is that the demand-signaling driven upregulation of NRT2.1 at the high nitrate side is largely abolished in the triple CK biosynthesis mutants (Poitout et al., 2018). Based on this we assumed that nitrate dependent CK signaling influences the efficiency of CEP based signaling (Figure 6A). This could occur either through affecting CEP stability, CEPR numbers or affinity, or even TCP20 effectiveness (see Guan et al., 2014).

To incorporate this into our model, we simulated that local nitrate presence leads to local cytokinin production, which is subsequently transported shootward, resulting in a systemic cytokinin pool (*CK*_*s*_, Eqs 7–8, Figure 6B). We subsequently redefined the CEP dependent growth control (Eq. 11a) as:

(15a) $$f_{\text{CEP}}=1+a_{\text{CEP}}\,\frac{{\left[{\text{CEP}}_{s}\right]}^{2}}{{\left[{\text{CEP}}_{s}\right]}^{2}+{\text{K}}_{\mathrm{NRT2}\,.1,\mathrm{CEP}}^{2}}*g_{N_{E}}*g_{\text{CK}}\,\text{with}$$

(15b) $$g_{\text{CK}}=a_{\text{CK}}+\left(1-a_{\text{CK}}\right)\,\frac{{\left[{\text{CK}}_{s}\right]}^{2}}{{\left[{\text{CK}}_{s}\right]}^{2}+K_{\mathrm{CEP},\mathrm{CK}}^{2}}\,\left(\text{Figure 6C}\right)$$

where *a*_CK_ is the CK independent and 1−*a*_CK_ the CK dependent fraction of CEP signaling, and *K*_*CEP,CK*_ is the CK level at which the CK dependent fraction of CEP signaling is at the half of its maximum level. This causes the fraction1−*a*_CK_ of CEP dependent growth stimulation to depend on both local nitrate presence as well as systemic CK signaling.

An important question is why plants, in addition to a “local nitrate presence signal” (*g*_*N_E*_), also would use a “systemic nitrate supply signal” (*g*_CK_). We hypothesized that while the local presence signal may serve to identify where to perform the preferential foraging growth, the systemic supply signal may serve to identify the extent of preferential foraging that is required by weighing supply and demand signals against one another. To investigate this, we simulated an “unbalanced” situation in which we partitioned the root system into a total of *n=16* compartments, with only one of these compartments being exposed to high, and all others being exposed to low nitrate levels. This way, we simulated a root system exposed to a single high nitrate patch (Figure 6D).

In Figure 6E we plotted the cumulative root length in the single compartment experiencing high nitrate level as well as the summed cumulative root length in all other compartments experiencing low nitrate levels. We did this for our previous model settings with CK-independent CEP signaling (Eq. 10), and for our new model CK-dependent CEP signaling (Eq. 14). Initially, all root compartments had an equal size, causing the summed length of low nitrate compartments to initially be 15 times higher than that of the single high nitrate compartment. We observed that for both situations, due to the high production of CEP in 15 out of 16 root compartments, root growth was strongly stimulated in the single compartment with a high nitrate level. Still, when taking into account CK signaling, growth promotion in the high nitrate patch was substantially less pronounced, and resulted in considerably less growth reduction in the other compartments.

In Figure 6F we show a standard split root experiment, now with CEP signaling being CK dependent. Compared to Figure 5F results were highly similar. Thus, if one half of the root system experiences high and one half experiences low nitrate levels, CK supply and CEP demand signals were quantitatively balanced. As a consequence, the additional requirement for CK signaling hardly affected root growth. If instead demand by far outstrips supply, CK signaling prevented an excessive growth response at a single high nitrate patch, preventing the full collapse of root growth in other patches that may potentially reach other high nitrate patches (Figure 6D).

### Mutants

Mutations in NRT1.1, CEP signaling and CK production have all been shown to cause a significant reduction in preferential root foraging (Ruffel et al., 2011; Mounier et al., 2014; Tabata et al., 2014; Ohkubo et al., 2017; Poitout et al., 2018). It was based on their individual large effects that we assumed that these players affect preferential root foraging in a synergistic rather than additive fashion and let us to model their effects in a largely multiplicative manner. With all players in place we now tested whether mutations in either three indeed strongly reduces preferential root foraging.

To simulate a mutation in NRT1.1, we put *f*_local_ to a constant intermediate value of 0.6. Additionally, since NRT1.1 not only functions as a nitrate sensor but also a nitrate transporter we assumed that the high affinity transport (*up*_*1*_) is reduced by 20%. For mutations in CEP or CK signaling we put the production of CEP or CK to zero. In Table 3 we show the length of the root system on the low nitrate side, the high nitrate side and their difference for wildtype as well as *nrt1.1*, *cep* and *ck* mutant plants after 6 days of simulated growth.

**TABLE 3:** Effect of *in silico* mutations on preferential root foraging.

**Plant**	**Length low N side**	**Length high N side**	**Difference**
WT	36	128	92
*nrt1.1*	51	77	26
*cep*	39	96	57
*ck*	39	101	62

We see that all three mutants resulted in a significant reduction of differences in root length between low and high nitrate sides of the root, as one would expect for a decrease in preferential root foraging. Additionally, *cep* and *ck* mutants showed less decrease in the reduction of root growth on the low nitrate side as compared to the *nrt1.1* mutant. This is consistent with the fact that CEP and CK signaling are involved only in the demand dependent enhancement of root growth on the high nitrate side, whereas NRT1.1 is involved in both repressing root growth for low nitrate and stimulating root growth for high nitrate. We did note that under our current model settings *cep* and *ck* mutants have a less strong effect on preferential foraging than the *nrt1.1* mutant. Importantly, the model we constructed is highly simplified and for example does not incorporate regulatory changes in transporter levels. Thus, an exact quantitative correspondence may not be reasonable to expect. Still, the fact that qualitatively the model reproduced all three mutants correctly strongly supports the validity of our modeling approach.

## Discussion

Preferential foraging of roots in nutrient rich soil patches is an important determinant of overall plant growth and fitness, enabling plants to survive in spatio-temporally varying conditions. Still, how exactly this preferential root foraging arises from the intricate network of internal and external nutrients sensing, signaling and subsequent responses has remained largely unclear. In the current study we investigated the case of preferential foraging for nitrate.

We used a highly simplified framework for modeling root system growth in which we incrementally incorporated different known aspects of nitrate dependent root growth regulation. Specifically, we incorporated local external nitrate dependent growth stimulation and repression involving among other molecular mechanisms the nitrate dependent auxin transport of NRT1.1 (Krouk et al., 2010; Mounier et al., 2014). We also incorporated demand driven growth stimulation involving CEP/CEPR/CEPD1/CEPD2 signaling and NRT2.1 upregulation (Tabata et al., 2014; Ohkubo et al., 2017), as well as systemic nitrate status dependent survival, foraging and suppression responses (Giehl and Von Wirén, 2014). Additionally, we incorporated intra-root system competition for carbon allocation. Finally, we included CK mediated supply signaling that influences the efficiency of the CEP demand signaling. Our model correctly reproduced the strong reduction in preferential root foraging upon mutations in either NRT1.1, CEP signaling or CK signaling.

Our model outcomes suggest that preferential nitrate foraging does not merely involve demand and supply signaling. Instead, enhanced root growth at the high nitrate side of a plant experiencing low nitrate at the other side also involves a reduced systemic repression compared to homogeneous high nitrate conditions. Similarly, reduced root proliferation at the low nitrate side of a plant experiencing high nitrate at the other side, also arises from a reduced foraging response compared to homogeneous low nitrate conditions. Finally, our model indicates that competition for carbon resources may contribute to the asymmetry in root lengths under heterogeneous nitrate conditions, enhancing the reduction in root growth at the low nitrate side.

Based on the above, we suggest that in addition to comparing the high and low nitrate experiencing sides of a plant root system to plant roots experiencing on both sides high or low nitrate, comparisons should be extended to roots experiencing on both sides intermediate nitrate levels. We expect that this extension will help tease apart the effects of overall nutrient status dependent systemic repression and foraging from nitrate heterogeneity driven demand and supply signaling effects. Combining this with large scale transcriptomics analyses (see Ruffel et al., 2011; Canales et al., 2014; Poitout et al., 2018), will furthermore enhance our capacity to discern which players of the nitrate signaling network execute which of these different effects. Indeed, in the current model we implemented a single generalized nitrate pool reflecting plant overall nitrate status, yet actual plant nitrate status signaling occurs via a variety of signals. These signals range from nitrate itself (Wang et al., 2004; Ruffel et al., 2011), nitrate metabolites (Walch-Liu et al., 2006), nitrate dependent changes in shoot-root auxin transport (Walch-Liu et al., 2006), overall plant ABA levels (Signora et al., 2002), and carbon to nitrate ratio (Malamy and Ryan, 2001). An important open question is why plants use this variety of signals related to plant nitrate status. Answering this question requires further research into how precisely these different signals relate to plant nitrate status, the concentration ranges and time-scales on which they act, and their potential cross-talk. In addition to measurements of root architecture and transcriptomics at various nitrate concentrations and time points, this will require the profiling of shoot and root metabolite and hormone content.

A key outcome of our model is the suggested role for CK signaling in nitrate foraging. Previous studies have suggested a role for CK in nitrate demand signaling (Ruffel et al., 2011; Poitout et al., 2018). This interpretation was based on the observed collapse of preferential root length increase on the high nitrate side in CK biosynthesis mutants. However, given the nitrate dependent production of CK, a role for CK in nitrate supply signaling appears more natural (Takei et al., 2002). Based and the observed effect of nitrate dependent CK signaling on demand driven upregulation of NRT2.1 (Poitout et al., 2018), we propose that this CK signal modulates the strength of CEP driven demand signaling. Importantly, while the current article was under revision, Ota et al. (2020) demonstrated that CEPD1, CEPD2, as well as CEPD-like 2 are upregulated in the shoot in response to trans-Zeatin type CK, supporting our hypothesis. Our hypothesis implies that preferential foraging on the high nitrate side involves coordinated nitrate supply, demand and local presence signaling. Using our model, we subsequently demonstrated that a supply signal impacting the efficiency of demand signaling would enable the plant to tune its extent of preferential foraging based on the balance between supply in demand. This helps prevent excessive investments in localized root growth in situations where nitrate supply is spatially highly restricted and other parts of the root system should keep foraging for nitrate. To test our model prediction, we propose experiments in split root plants with a mildly high and a low nitrate side. If our predictions are correct, under normal conditions only moderate preferential foraging should occur, whereas after addition of CK this preferential foraging should increases because of the enhanced effectiveness of the demand signaling.

Complementary to the suggested extensions in experimental setup and analyses, elaborations of the modeling framework developed here will be essential to increase its predictive power. A first critical expansion will be to implement our model within a spatially explicit, branching root architecture such as typically used in FSP models (for example, Schnepf et al., 2018). A spatially explicit, growing and developing root architecture will enable taking into account important distinctions between the growth of main roots and lateral roots, growth of existing and formation of new lateral roots, and root type and developmental stage dependent nitrate signaling and responses. Another essential model extension will be to take into account the well-known effect of plant nitrate status on root-shoot growth ratios, and the effect this has on plant carbon production (Ericsson, 1995; Ågren and Franklin, 2003), as this will result in an additional feedback on root growth responses.

Other interesting directions for future work would be to attempt to extend the models explanatory power to the apparent sensitivity of root growth to temporal changes in nitrate levels (Shemesh et al., 2010a, b, 2011). This likely requires the incorporation of additional model components, such as vacuoles acting as internal nitrate stores, or the regulation of expression of nitrate transporters (Aibara and Miwa, 2014). On a similar note, considering the effects of neighboring plants competing for nitrate (Ljubotina and Cahill, 2019), will require at least the incorporation of soil nitrate dynamics (Robinson, 2001), and potentially plant–plant communication mechanisms.

## Data Availability Statement

The datasets generated for this study are available on request to the corresponding author.

## Author Contributions

MB developed the initial version of the model and wrote an early version of the manuscript. JS analyzed model output, created figures, and edited the manuscript. KT conceived the research and wrote the manuscript.

## Conflict of Interest

The authors declare that the research was conducted in the absence of any commercial or financial relationships that could be construed as a potential conflict of interest.

## Footnotes

1 http://bioinformatics.bio.uu.nl/khwjtuss/NitrateModel

## References

[B1] Ågren G. I. Franklin O. (2003). Root:shoot ratios, optimization and nitrogen productivity. *Ann. Bot.* 92 795–800. 10.1093/aob/mcg203 14565938 PMC4243620

[B2] Aibara I. Miwa K. (2014). Strategies for optimization of mineral nutrient transport in plants: multilevel regulation of nutrient-dependent dynamics of root architecture and transporter activity. *Plant Cell Physiol.* 55 2027–2036. 10.1093/pcp/pcu156 25378690

[B3] Angle J. S. Gross C. M. Hill R. L. McIntosh M. S. (1993). Soil nitrate concentrations under corn as affected by tillage, manure, and fertilizer applications. *J. Environ. Qual.* 147 141–147. 10.2134/jeq1993.00472425002200010018x

[B4] Araya T. Miyamoto M. Wibowo J. Suzuki A. Kojima S. Tsuchiya Y. N. (2014). CLE-CLAVATA1 peptide-receptor signaling module regulates the expansion of plant root systems in a nitrogen-dependent manner. *Proc. Natl. Acad. Sci. U.S.A.* 111 2029–2034. 10.1073/pnas.1319953111 24449877 PMC3918772

[B5] Boer M. D. Santos Teixeira J. Ten Tusscher K. H. (2019). Modeling of root nitrate responses suggests preferential foraging arises from the integration of demand, supply and local presence signals. *BioRxiv* [Preprint], 10.1101/2019.12.19.882548 PMC7268170 32536935

[B6] Canales J. Moyano T. C. Villarroel E. Gutiérrez R. A. (2014). Systems analysis of transcriptome data provides new hypotheses about *Arabidopsis* root response to nitrate treatments. *Front. Plant Sci.* 5:22 10.3389/fpls.2014.00022 PMC3917222 24570678

[B7] Cerezo M. Tillard P. Filleur S. Munos S. Daniel-Vedele F. Gojon A. (2001). Major alterations of the regulation of root no 3- uptake are associated with the mutation of Nrt2.1 and Nrt2.2 Genes in *Arabidopsis*. *Plant Physiol.* 127 262–271. 10.1104/pp.127.1.262 11553754 PMC117982

[B8] Crawford N. M. Glass A. D. M. (1998). Molecular and physiological aspects of nitrate uptake in plants. *Trends Plant Sci.* 3 389–395. 10.1016/S1360-1385(98)01311-9

[B9] Ericsson T. (1995). Growth and shoot: root ratio of seedlings in relation to nutrient availability. *Plant Soil* 169 205–214. 10.1007/BF00029330

[B10] Fujikake H. Yamazaki A. Ohtake N. Sueyoshi K. Matsuhashi S. Ito T. (2003). Quick and reversible inhibition of soybean root nodule growth by nitrate involves a decrease in sucrose supply to nodules. *J. Exp. Bot.* 54 1379–1388. 10.1093/jxb/erg147 12709484

[B11] Giehl R. F. H. Gruber B. D. Von Wirén N. (2014). It’s time to make changes: modulation of root system architecture by nutrient signals. *J. Ex. Bot.* 65 769–778. 10.1093/jxb/ert421 24353245

[B12] Giehl R. F. H. Von Wirén N. (2014). Root nutrient foraging. *Plant Physiol.* 166 509–517. 10.1104/pp.114.245225 25082891 PMC4213083

[B13] Girin T. El-Kafafi E.-S. Widiez T. Erban A. Hubberten H.-M. Kopka J. (2010). Identification of *Arabidopsis Mutants* impaired in the systemic regulation of root nitrate uptake by the nitrogen status of the plant. *Plant Physiol.* 153 1250–1260. 10.1104/pp.110.157354 20448103 PMC2899898

[B14] Gruber B. D. Giehl R. F. H. Friedel S. Von Wirén N. (2013). Plasticity of the *Arabidopsis* root system under nutrient deficiencies. *Plant Physiol.* 163 161–179. 10.1104/pp.113.218453 23852440 PMC3762638

[B15] Guan P. (2017). Dancing with hormones: a current perspective of nitrate signaling and regulation in *Arabidopsis*. *Fronti. Plant Sci.* 8:1697 10.3389/fpls.2017.01697 PMC5625010 29033968

[B16] Guan P. Wang R. Nacry P. Breton G. Kay S. A. Pruneda-paz J. L. (2014). Nitrate foraging by *Arabidopsis* roots is mediated by the transcription factor TCP20 through the systemic signaling pathway. *Proc. Natl. Acad. Sci. U.S.A.* 111 15267–15272. 10.1073/pnas.1411375111 25288754 PMC4210337

[B17] Hellebrand H. J. Scholz V. Kern J. Kavdir Y. (2005). N2O release during cultivation of energy crops. *Agrartech. Forsch.* 11 114–124.

[B18] Jin Z. Zhu Y. Li X. Dong Y. An Z. (2015). Soil N retention and nitrate leaching in three types of dunes in the Mu Us desert of China. *Sci. Rep.* 5 1–8. 10.1038/srep14222 PMC4642556 26370253

[B19] Krouk G. Lacombe B. Bielach A. Perrine-Walker F. Malinska K. Mounier E. (2010). Nitrate-regulated auxin transport by NRT1.1 defines a mechanism for nutrien sensing in plants. *Dev. Cell* 18 927–937. 10.1016/j.devcel.2010.05.008 20627075

[B20] Lally D. Ingmire P. Tong H.-Y. He Z.-H. (2001). Antisense expression of a cell wall-associated protein kinase, WAK4, inhibits cell elongation and alters morphology. *Plant Cell* 13 1317–1331. 10.1105/tpc.13.6.1317 11402163 PMC135583

[B21] Little D. Y. Rao H. Oliva S. Daniel-Vedele F. Krapp A. Malamy J. E. (2005). The putative high-affinity nitrate transporter NRT2.1 represses lateral root initiation in response to nutritional cues. *Proc. Natl. Acad. Sci. U.S.A.* 102 13693–13698. 10.1073/pnas.0504219102 16157886 PMC1224627

[B22] Ljubotina M. K. Cahill J. F. Jr. (2019). Effects of neighbour location and nutrient distributions on root foraging behaviour of the common sunflower. *Proc. R. Soc. B* 286:955 10.1098/rspb.2019.0955 PMC6784730 31530149

[B23] Ma W. Li J. Qu B. He X. Zhao X. Li B. (2014). Auxin biosynthetic gene TAR2 is involved in low nitrogen-mediated reprogramming of root architecture in *Arabidopsis*. *Plant J.* 78 70–79. 10.1111/tpj.12448 24460551

[B24] Malamy J. E. Ryan K. S. (2001). Environmental regulation of lateral root initiation in *Arabidopsis*. *Plant Physiol.* 127 899–909. 10.1104/pp.010406 11706172 PMC129261

[B25] Marcelis L. F. M. (1996). Sink strength as a determinant of dry matter partitioning in the whole plant. *J. Exp. Bot.* 47 1281–1291. 10.1093/jxb/47.Special_Issue.1281 21245260

[B26] Mounier E. Pervent M. Ljung K. Gojon A. Nacry P. (2014). Auxin-mediated nitrate signalling by NRT1.1 participates in the adaptive response of *Arabidopsis* root architecture to the spatial heterogeneity of nitrate availability. *Plant Cell Environ.* 37 162–174. 10.1111/pce.12143 23731054

[B27] Naz M. Luo B. Guo X. Chen J. Fan X. (2019). Overexpression of nitrate transporter OsNRT2.1 enhances nitrate-dependent root elongation. *Genes* 10 1–18. 10.3390/genes10040290 PMC6523718 30970675

[B28] Ohkubo Y. Tanaka M. Tabata R. Ogawa-Ohnishi M. Matsubayashi Y. (2017). Shoot-to-root mobile polypeptides involved in systemic regulation of nitrogen acquisition. *Nat. Plants* 3 1–6. 10.1038/nplants.2017.29 28319056

[B29] Okamoto Y. Suzuki T. Sugiura D. Kiba T. Sakakibara H. Hachiya T. (2019). Shoot nitrate underlies a perception of nitrogen satiety to trigger local and systemic signaling cascades in *Arabidopsis thaliana*. *Soil Sci. Plant Nutr.* 65 56–64. 10.1080/00380768.2018.1537643

[B30] Ota R. Ohkubo Y. Yamashita Y. Ogawa-Ohnishi M. Matsubayashi Y. (2020). Shoot-to-root mobile CEPD-like 2 integrates shoot nitrogen status to systemically regulate nitrate uptake in *Arabidopsis*. *Nat. Commun.* 11 1–9. 10.1038/s41467-020-14440-8 32005881 PMC6994653

[B31] Poitout A. Crabos A. Petøík I. Novák O. Krouk G. Lacombe B. (2018). Responses to systemic nitrogen signaling in *Arabidopsis* roots involve trans-zeatin in shoots. *Plant Cell* 30 1243–1257. 10.1105/tpc.18.00011 29764985 PMC6048791

[B32] Remans T. Nacry P. Pervent M. Filleur S. Diatloff E. Mounier E. (2006). The Arabidopsis NRT1.1 transporter participates in the signaling pathway triggering root colonization of nitrate-rich patches. *Proc. Natl. Acad. Sci. U.S.A.* 103 19206–19211. 10.1073/pnas.0605275103 17148611 PMC1748200

[B33] Robinson D. (2001). Root proliferation, nitrate inflow and their carbon costs during nitrogen capture by competing plants in patchy soil. *Plant Soil* 232 41–50. 10.1023/A:101377818094

[B34] Ruffel S. Krouk G. Ristova D. Shasha D. Birnbaum K. D. Coruzzi G. M. (2011). Nitrogen economics of root foraging: transitive closure of the nitrate-cytokinin relay and distinct systemic signaling for N supply vs. demand. *Proc. Natl. Acad. Sci. U.S.A.* 108 18524–18529. 10.1073/pnas.1108684108 22025711 PMC3215050

[B35] Sasse J. Jordan J. S. DeRaad M. Whiting K. Zhalnina K. Northen T. (2019). Root morphology and exudate availability is shaped by particle size and chemistry in *Brachypodium distachyon*. *BioRxiv* [Preprint], 10.1101/651570 PMC7330624 32642632

[B36] Schnepf A. Leitner D. Landl M. Lobet G. Mai T. H. Morandage S. (2018). CRootBox: a structural-functional modelling framework for root systems. *Ann. Bot.* 121 1033–1053. 10.1093/aob/mcx221 29432520 PMC5906965

[B37] Shemesh H. Arbiv A. Gersani M. Ovadia O. Novoplansky A. (2010a). The effects of nutrient dynamics on root patch choice. *PLoS One* 5:824 10.1371/journal.pone.0010824 PMC2877079 20520811

[B38] Shemesh H. Ovadia O. Novoplansky A. (2010b). Anticipating future conditions via trajectory sensitivity. *Plant Signal. Behav.* 5 1501–1503. 10.4161/PSB5.11.13660 21057218 PMC3115267

[B39] Shemesh H. Rosen R. Eshel G. Novoplansky A. Ovadia O. (2011). The effect of steepness of temporal resource gradients on spatial root allocation. *Plant Signal. Behav.* 6 1356–1360. 10.6141/psb.6.9.16444 22019637 PMC3258065

[B40] Signora L. De Smet I. Foyer C. H. Zhang H. (2002). ABA plays a central role in mediating the regulatory effects of nitrate on root branching in *Arabidopsis*. *Plant J.* 28 655–662. 10.1046/j.1365-313x.2001.01185.x 11851911

[B41] Sun H. Tao J. Bi Y. Hou M. Lou J. Chen X. (2018). OsPIN1b is involved in rice seminal root elongation by regulating root apical meristem activity in response to low nitrogen and phosphate. *Sci. Rep.* 8 1–11. 10.1038/s41598-018-29784-x 30158652 PMC6115472

[B42] Tabata R. Sumida K. Yoshii T. Ohyama K. Shinohara H. Matsubayashi Y. (2014). Perception of root-derived peptides by shoot LRR-RKs mediates systemic N-demand signaling. *Science* 346 343–346. 10.1126/science.1257800 25324386

[B43] Takei K. Takahashi T. Sugiyama T. Yamaya T. Sakakibara H. (2002). Multiple routes communicating nitrogen availability from roots to shoots: a signal transduction pathway mediated by cytokinin. *J. Exp. Bot.* 53 971–977. 10.1093/jexbot/53.370.971 11912239

[B44] Taylor A. A. De-Felice J. Havill D. C. (1982). Seasonal variation in nitrogen availability and utilization in an acidic and calcareous soil. *New Phytol.* 92 141–152. 10.1111/j.1469-8137.1982.tb03370.x

[B45] Terasaka K. Blakeslee J. J. Titapiwatanakun B. Peer W. A. Bandyopadhyay A. Makam S. N. (2005). PGP4, an ATP binding cassette p-glycoprotein, catalyzes auxin transport in arabidopsis thaliana roots. *Plant Cell* 17 2922–2939. 10.1105/tpc.105.035816 16243904 PMC1276020

[B46] van de Sande-Bakhuyzen H. L. (1928). Studies upon wheat grown under constant conditions. *Plant Physiol.* 3 7–30. 10.1104/pp.3.1.1 16652551 PMC441344

[B47] Vidal E. A. Álvarez J. M. Gutiérrez R. A. (2014). Nitrate regulation of AFB3 and NAC4 gene expression in Arabidopsis roots depends on NRT1.1 nitrate transport function. *Plant Signal. Behav.* 9 1–4. 10.4161/psb.28501 PMC4091544 24642706

[B48] Vidal E. A. Araus V. Lu C. Parry G. Green P. J. Coruzzi G. M. (2010). Nitrate-responsive miR393/AFB3 regulatory module controls root system architecture in *Arabidopsis thaliana*. *Proc. Natl. Acad. Sci. U.S.A.* 107 4477–4482. 10.1073/pnas.0909571107 20142497 PMC2840086

[B49] Vidal E. A. Moyano T. C. Riveras E. Contreras-López O. Gutiérrez R. A. (2013). Systems approaches map regulatory networks downstream of the auxin receptor AFB3 in the nitrate response of *Arabidopsis thaliana* roots. *Proc. Natl. Acad. Sci. U.S.A.* 110 12840–12845. 10.1073/pnas.1310937110/23847199 PMC3732920

[B50] Walch-Liu P. Ivanov I. I. Filleur S. Gan Y. Remans T. Forde B. G. (2006). Nitrogen regulation of root branching. *Ann. Bot.* 97 875–881. 10.1093/aob/mcj601 16339770 PMC2803407

[B51] Wang R. Tischner R. Gutiérrez R. A. Hoffman M. Xing X. Chen M. (2004). Genomic analysis of the nitrate response using a nitrate reductase-null mutant of *Arabidopsis*. *Plant Physiol.* 136 2512–2522. 10.1104/pp.104.044610 15333754 PMC523318

[B52] Weil R. R. Brady N. C. (1996). *The Nature and Properties of Soil.* London: Pearson.

[B53] Xu P. Cai W. (2019). Nitrate-responsive OBP4-XTH9 regulatory module controls lateral root development in *Arabidopsis thaliana*. *PLoS Genet.* 15:e08465 10.1371/journal.pgen.1008465 PMC6821136 31626627

